# FDA-approved drugs as potential covalent inhibitors of key SARS-CoV-2 proteins: an in silico approach

**DOI:** 10.55730/1300-0152.2741

**Published:** 2025-04-07

**Authors:** Murat SERİLMEZ, Anwar ABUELRUB, Ismail EROL, Serdar DURDAĞI

**Affiliations:** 1 Department of Basic Oncology, Oncology Institute, Istanbul University, İstanbul, Turkiye; 2 Computational Drug Design Center (HİTMER), Bahçeşehir University, İstanbul, Turkiye; 3 Department of Analytical Chemistry, School of Pharmacy, Bahçeşehir University, İstanbul, Turkiye; 4 Department of Pharmaceutical Chemistry, School of Pharmacy, Bahçeşehir University, İstanbul, Turkiye

**Keywords:** COVID-19, covalent docking, molecular dynamics simulation, molecular mechanics-generalized Born surface area

## Abstract

**Background/aim:**

The COVID-19 pandemic caused by SARS-CoV-2 necessitated rapid development of effective therapeutics, prompting this study to identify potential inhibitors targeting key viral and host proteins: RNA-dependent RNA polymerase (RdRp), main protease (Mpro), transmembrane serine protease 2 (TMPRSS2), and angiotensin-converting enzyme 2 (ACE2).

**Methods:**

We used covalent docking and molecular dynamics (MD) simulations to screen FDA-approved compounds against these targets using diverse covalent reaction mechanisms. Top-ranking compounds underwent further evaluation through MD simulations to assess binding stability and conformational dynamics.

**Results:**

Several promising drug repurposing candidates were identified: bremelanotide, lanreotide, histrelin, and leuprolide as potential RdRp inhibitors; azlocillin, cefiderocol, and sultamicillin for Mpro inhibition; tenapanor, isavuconazonium, and ivosidenib targeting TMPRSS2; and cefiderocol, cefoperazone, and ceftolozane as potential ACE2 inhibitors.

**Conclusion:**

This study provides valuable insights into repurposing existing drugs as potential COVID-19 therapeutics by targeting crucial viral proteins. However, further experimental validation and preclinical studies are necessary to confirm the efficacy and safety of these compounds before consideration for clinical application.

## 1. Introduction

The COVID-19 pandemic caused by the SARS-CoV-2 coronavirus is still catalyzing an unprecedented worldwide effort to develop lifesaving treatments. Since the emergence of the virus in late 2019, there have been considerable efforts across the globe to develop effective drugs and vaccines to combat this formidable disease (Baden et al., 2021), culminating in the rapid development and deployment of several promising therapeutics and vaccines. Remdesivir is one such therapeutic that aims to alleviate symptoms, reduce disease severity, and prevent complications in infected individuals. In general, antiviral drugs have shown promise for shortening illness duration and improving recovery rates in hospitalized patients. In addition, monoclonal antibodies have been authorized for emergency use to neutralize the virus and prevent progression to severe disease, especially in high-risk groups (Beigel et al., 2020). Primarily for more severe cases, multiple vaccine candidates were produced, such as mRNA, viral vectors, protein subunits, and inactivated viruses. A number of vaccines have been made available that have shown remarkable effectiveness in preventing COVID-19 infection and reducing illness severity, including those from Pfizer-BioNTech, AstraZeneca, Moderna, and Johnson & Johnson (Polack et al., 2020). However, challenges persist with vaccines, especially in vaccine distribution, hesitancy to receive vaccines, and the introduction of novel variations, posing significant obstacles in the global effort to control the pandemic (Voysey et al., 2021).

Human coronaviruses, particularly NL63, OC43, 229E, and HKU1 variants, are associated with mild respiratory illnesses, usually resembling the common cold. However, in recent years, more severe strains have emerged, notably the severe acute respiratory syndrome-related coronavirus (SARS-CoV), SARS-CoV-2, and the Middle East respiratory syndrome coronavirus (MERS-CoV) that contribute to the ongoing COVID-19 epidemic. These pathogens pose significant threats to morbidity and mortality rates related to severe respiratory syndromes (Perlman and Netland, 2009). The life cycle of coronaviruses is driven by a multitude of intricate proteins, including main protease (Mpro)—an essential enzyme for processing viral polyproteins into functional units that are required for replication (Zumla et al., 2016). Spike (S) protein is the key mediator of viral entry into host cells and facilitates binding to the angiotensin-converting enzyme 2 (ACE2) host cell receptor (Hoffmann et al., 2020). RNA-dependent RNA polymerase (RdRp) is an essential enzyme involved in viral replication (Wang et al., 2020). Additionally, helicase (nsp13) is an enzyme that plays a vital role in unwinding virus RNA during replication. Targeting these essential proteins with small molecules provides promising avenues for developing antiviral therapeutics to defeat COVID-19 and other related coronavirus diseases (Adedeji and Sarafianos, 2014).

The coronavirus entry process of SARS-CoV and SARS-CoV-2 critically depends on the S protein that consists of two functional subunits: S1 and S2. These subunits work in concert to facilitate viral attachment and membrane fusion. The S1 subunit initiates the process by binding to the ACE2 receptor on host cells (Lan et al., 2020) triggering conformational changes that expose the receptor-binding domain (RBD). Following attachment, the S2 subunit mediates the fusion between viral and cellular membranes, allowing the virus to enter the host cell (Walls et al., 2020; Bosch et al., 2003). This process is further facilitated by the host protease transmembrane serine protease 2 (TMPRSS2) that primes and cleaves the S protein upon receptor binding to enhance the fusion process. This proteolytic activation is crucial for viral entry and pathogenesis (Hoffmann et al., 2020; Shulla et al., 2011). Additionally, viral polyproteins are cleaved into essential proteins required for replication by the 3C-like protease Mpro (Zhang et al., 2020). Inhibiting Mpro leads to the disruption of viral replication. Furthermore, viral transcripts and genomes are synthesized by RdRp, which is crucial for replication and protein synthesis (Gordon et al., 2020; Jin et al., 2020; Wang et al., 2020). Inhibiting RdRp with drugs like remdesivir halts viral replication, offering potential treatments against coronaviruses (Yin et al., 2020).

The repurposing search for therapeutically potent drugs against coronaviruses benefits significantly from covalent docking approaches when targeting viral proteins including Mpro, RdRp, and other essential viral components. This strategy can prove advantageous in determining the inhibitors that establish permanent chemical bonds with their targets, resulting in stable molecular interactions that persist over extended periods. These covalent interactions allow for the design of inhibitors with enhanced binding affinity to target proteins, potentially leading to greater potency and prolonged inhibition of viral replication. Additionally, covalent inhibitors can offer improved selectivity and reduced susceptibility to drug resistance compared to noncovalent inhibitors (Jin et al., 2020). Jin et al. (2020) demonstrated the effectiveness of covalent inhibitors targeting Mpro of SARS-CoV-2. By employing covalent docking, the researchers identified several α-ketoamide compounds that formed covalent bonds with the active site cysteine residue of Mpro, leading to potent inhibition of viral replication. These findings highlight the utility of covalent docking in the design of therapeutically active drugs against coronaviruses (Jin et al., 2020).

## 2. Materials and methods

### 2.1. Molecular preparation

A total of 2360 small-molecule compounds approved by the FDA were obtained from the DrugBank database and prepared using the LigPrep package available in the Schrödinger Maestro software^1^. These molecules were adjusted to a neutral pH state with ionization at 7.4 using the Epik module (Rostkowski et al., 2011) and the OPLS3e force field (Harder et al., 2016).

### 2.2. Protein structure preparation

The 3D structures of the proteins of interest were obtained from the Protein Data Bank (PDB). In the case of Mpro, the crystal structure with the PDB code 7CWC was retrieved (Durdagi et al., 2021). For the S/ACE-2 complex, the structure with PDB code 6M0J was retrieved (Lan et al., 2020). For the RdRp, the structure with PDB code 6M71 was retrieved (Gao et al., 2020). Due to the absence of an experimentally derived crystal structure for TMPRSS2 in the PDB, we utilized a homology model sourced from our earlier study (Durdağı, 2020). This three-dimensional structural model served as the foundation for our virtual screening investigations. The Protein Preparation module in Schrödinger Maestro software^1^ was used for protein structure preparation. Hydrogen atoms were added by determining the bond orders. Disulfide and metal bonds were formed. Water molecules within 5 Å of the binding site were retained, while other water molecules were eliminated. PROPKA software (Rostkowski et al., 2011) was used to ascertain the protonation status of the amino acid residues. OPLS3e force field (Harder et al., 2016) was used for restricted minimization, with a heavy atom convergence threshold of 0.3 Å.

### 2.3. Covalent docking

Covalent docking was performed using the CovDock tool (Zhu et al., 2014) within the Maestro molecular modeling suite. CovDock specifies the best covalent complexes using the Prime energy model, which has undergone thorough validation, and an affinity score that encapsulates the essential components of effective covalent docking. The workflow begins with Glide docking on the target structure, where the reactive residue is temporarily converted into Alanine residue. Next, the reactive residue is reintroduced. In order to create a covalent link, the ligand is sampled in several positions. For the purpose of determining which covalent complexes have the optimal score, the covalent complexes are reduced using the Prime VSGB2.0 energy model. GLU166, CYS145, and HIS41 comprise the central catalytic region of Mpro. For the covalent docking of ligands to the Mpro receptor, the x, y, and z coordinates 20.9, −0.6, and −28.2 were used to define the binding site in the covalent docking module, respectively. The Mpro protein underwent various types of reactions, including disulfide formation, phosphonate participation, betalactam participation, double and triple bond inclusion, nucleophilic substitution, boronic acid addition, epoxide opening, conjugated participation for alkene (activated nitrile), conjugated addition for alkyl (activated aryl), and conjugated participation for alkene (activated nitrile). The catalytic site of TMPRSS2 comprises the amino acids HIS296, GLU345, and SER441. For the covalent docking of ligands to the TMPRSS2 receptor, the x, y, and z coordinates 10.02, −0.78, and 37.92 were used to define the binding site in the covalent docking module, respectively. The following types of covalent reactions were considered for the docking process: nucleophilic addition (double bond, triple bond, and nucleophilic substitution), Michael addition, boronic acid addition, epoxide opening, phosphonate addition, betalactam addition for TMPRSS2, conjugated addition for alkene (activated nitrile), conjugated addition for alkyl (activated carbonyl), and conjugated addition for alkyl (activated aryl). After conducting the covalent docking calculations, the results were analyzed based on the binding interactions and scores.

With regard to the SARS-CoV-2 S protein interacting with ACE2, the catalytic site comprises TYR449, LYS493, SER494, and ARG498, while the ACE2 binding site includes ASP38, SER47, and TYR41. The x, y, and z coordinates to specify the binding site for covalent docking of ligands to the S/ACE2 complex were 13.02, 4.7, and 8.1, respectively. For the SARS-CoV-2 RdRp, the catalytic site consists of CYS622, ASP623, and THR687, and the x, y, and z coordinates used for the covalent docking binding site were 116.6, 121.5, and 127.6, respectively. The following types of covalent reactions were considered during the docking process for both S/ACE2 and RdRp: nucleophilic addition (double bond, triple bond, and nucleophilic substitution), Michael addition, boronic acid addition, epoxide opening, phosphonate addition, betalactam addition, conjugated addition for alkene (activated nitrile), conjugated addition for alkyl (activated carbonyl), conjugated addition for alkyl (activated aryl), and disulfide formation.

### 2.4 Molecular dynamics (MD) simulations

The top-ranked complexes were placed in orthorhombic simulation boxes with a 10 Å buffer distance to ensure adequate solvation and system stability solved by TIP3P water models and neutralized by adding counterions of NaCl solution at 0.15 M. All-atom MD simulations were performed in the Desmond software. Prior to simulation runs, the systems had been in equilibrium using the default relaxation steps in Desmond. Using the NPT ensemble, Nose-Hoover thermostat, and Martyna-Tobias-Klein barostat, the simulations were carried out at a physiological temperature of 310 K and a constant pressure of 1.01325 bar. The smooth particle mesh Ewald technique was applied to calculate long-range electrostatic interactions with periodic boundary conditions, and a 9 Å cutoff was applied for Lennard-Jones interactions and short-range electrostatics. The multistep integrator RESPA was used, with different time intervals for bonded (2 fs) and nonbonded (6 fs) interactions. The OPLS3e force field was used. Moreover, the VSGB 2.0 solvation model was used to compute the average molecular mechanics-generalized Born surface area (MMGBSA) binding free energy across 200 frames.

For root mean square deviation (RMSD) analysis, explicit stability criteria were established to ensure reproducibility. Specifically, molecular systems were considered stable when RMSD values did not deviate beyond 4 Å from the initial position of the structure. This threshold was applied consistently across all simulated systems to standardize stability assessments and enable meaningful comparisons between different ligand-protein complexes. In this study, LigFitProt RMSD was used which measures the RMSD of ligand atoms after aligning the protein backbones.

## 3. Results and discussion

Although all reaction types described in the Methods section were conducted for each target structure, only the results of the reactions yielding the top-docking scores are presented in the Results section.

### 3.1. Computational analysis identified the nucleophilic addition to a double bond reaction as a key interaction for potential RdRp inhibitors

**Figure 1:**
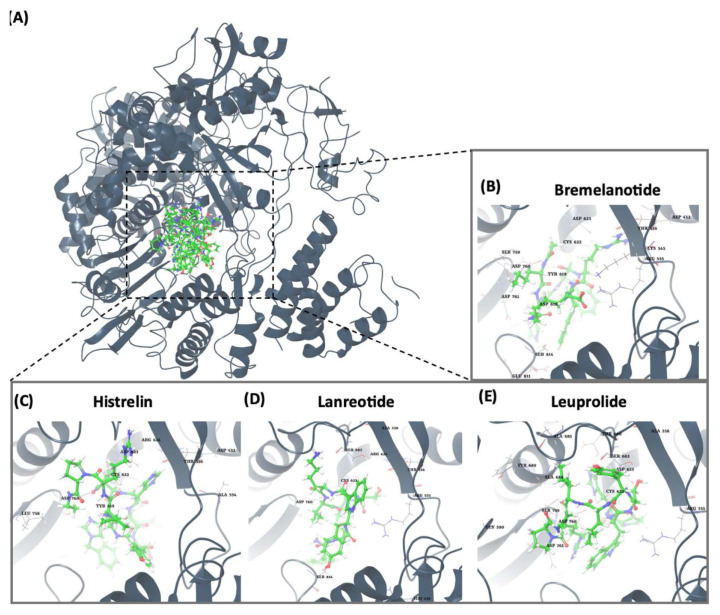
(A) The orientation of top-scored small molecules in the binding pocket of RdRp during a nucleophilic addition to a double bond reaction, involving (B) bremelanotide, (C) histrelin, (D) lanreotide, and (E) leuprolide.

The top four molecules and the residues involved in the interaction between each molecule and the RdRp of the SARS-CoV-2 virus are summarized in Table 1 and Figure 1. The docking and average MMGBSA scores are reported in kcal/mol, where lower values indicate stronger binding affinities. Among the four molecules, bremelanotide had the lowest docking score of −8.617 kcal/mol and a free binding energy MMGBSA of −96.6 kcal/mol, suggesting a strong binding affinity towards RdRp. The key residues involved in the hydrogen bond interaction between bremelanotide and RdRp, as shown in Figure S1, include ASP452, LYS545, TYR619, ASP623, SER759, and GLU811. ARG555, ASP760, and ASP761 had H-bonds and water bridge interactions. A hydrophobic interaction was observed with the CYS622 residue, while ASP618 was involved in ionic and water bridge interactions. Besides, THR556 and SER814 showed water bridge interactions. These residues likely play a crucial role in the binding process and act as a potential inhibitor for RdRp. The second-lowest docking score observed was lanreotide (−7.714 kcal/mol) with an MMGBSA score of −87.4 kcal/mol, indicating a relatively strong binding affinity. Furthermore, the hydrogen bond interactions between lanreotide and RdRp, illustrated in Figure S2, involved the residues THR556, ALA558, ARG624, and SER682. A hydrophobic interaction was observed with CYS622. ARG555, ASP760, and SER814 had more than one interaction type, including hydrogen bonding and water bridges. Histrelin had a higher docking score of −6.465 kcal/mol with a −94.5 MMGBSA score, suggesting strong binding affinities. Figure S3 shows that ASP623 was involved in the hydrogen bond interaction with histrelin, as well as the hydrophobic interaction with CYS622. ASP452, ALA554, THR556, TYR619, CYS622, ASP623, SER759, and ASP760 had H-bond and water bridge interactions. Leuprolide had a docking score of −5.788 kcal/mol and a MMGBSA value of −140.2. Figure S4 shows that leuprolide interacts through hydrogen bonds with GLY590 and SER682. Hydrophobic interactions were found with CYS622, ALA685, and ALA688. ARG555 and TYR689 had both hydrophobic and water-bridge interactions. ARG569 had ionic interaction. ALA558, ASP623, and THR680 had water bridges. Moreover, SER759, ASP760, and ASP761 had both hydrogen bonding and water bridge interactions. It is noteworthy that several residues, such as ARG555, CYS622, ASP623, and ASP760, were common among the interactions of multiple molecules with RdRp, suggesting their potential importance in the binding and inhibition mechanisms. Overall, the results indicate that these top four molecules had strong binding affinities towards RdRp based on their docking scores, making them promising candidates for further investigation as potential inhibitors of the COVID-19 virus replication process.

Figure S5 shows the RMSD of backbone atoms for protein-ligand complexes over time for the nucleophilic addition to a bond reaction. It illustrates that leuprolide values (in green) fluctuate around 2 to 3 Å throughout the simulation time, indicating relatively moderate structural deviations from the initial target protein conformation.Bremelanotide-complex (in orange) among the four molecules had relatively stable RMSD values around 2 to 3 Å throughout the simulations. Moreover, the RMSD values of histrelin (in light blue) are generally lower compared to the other molecules and mostly stable at 2 Å. However, it starts to increase after 73 ns to reach 3.5 Å. This indicates that histrelin-complex maintains a relatively stable conformation throughout the simulation, with minimal structural deviations. Lanreotide-complex (in red) RMSD values showed a few instances at the beginning of the simulations between 0 and 30 ns where the RMSD reached approximately 4 Å, but after that, it stabilized between 2 and 3.5 Å for the remainder of the simulation time. This suggests that lanreotide-complex undergoes more significant structural changes and deviations from its initial conformation during the simulation. In summary, leuprolide, bremelanotide, and histrelin-bound targets had the lowest RMSD values, suggesting the highest structural stability. Lanreotide showed moderate deviation and stability with the highest RMSD values, indicating more significant conformational changes during the simulation.

Several proteins are related to the SARS-CoV-2 virus. These proteins play various roles in the life cycle of the virus and interaction with the host. In our study, we targeted the most prominent proteins, including RdRp, TMPRSS2, Mpro, and ACE2. The top molecules identified for RdRp in the nucleophilic addition to a double bond reaction emerged as promising inhibitors targeting SARS-CoV-2 RdRp, offering potential therapeutic avenues against COVID-19. Bremelanotide, which is a peptide analog of α-melanocyte-stimulating hormone, is mostly used to treat hypoactive sexual desire disorder (HSDD) in premenopausal women (Edinoff et al., 2022). However, in this study, bremelanotide showed a potential antiviral property by inhibiting RdRp activity. Likewise, lanreotide is a somatostatin analog primarily used in neuroendocrine tumor therapy (Frankel et al., 2021). Histrelin and leuprolide have been widely used for central precocious puberty treatments (Lee et al., 2012; Klein et al., 2016; Silverman et al., 2021). However, our study found another potential activity for this drug by targeting RdRp. This enzyme is considered one of the most prominent proteins related to SARS-CoV-2 virus RNA replication. These compounds hold significant potential for combating SARS-CoV-2 by targeting a key enzyme essential for viral replication. Further research and clinical trials are warranted to validate their efficacy and safety profiles in COVID-19 treatment.

### 3.2. In silico analysis reveals nucleophilic addition as a key interaction for potential TMPRSS2 inhibitors

**Figure 2:**
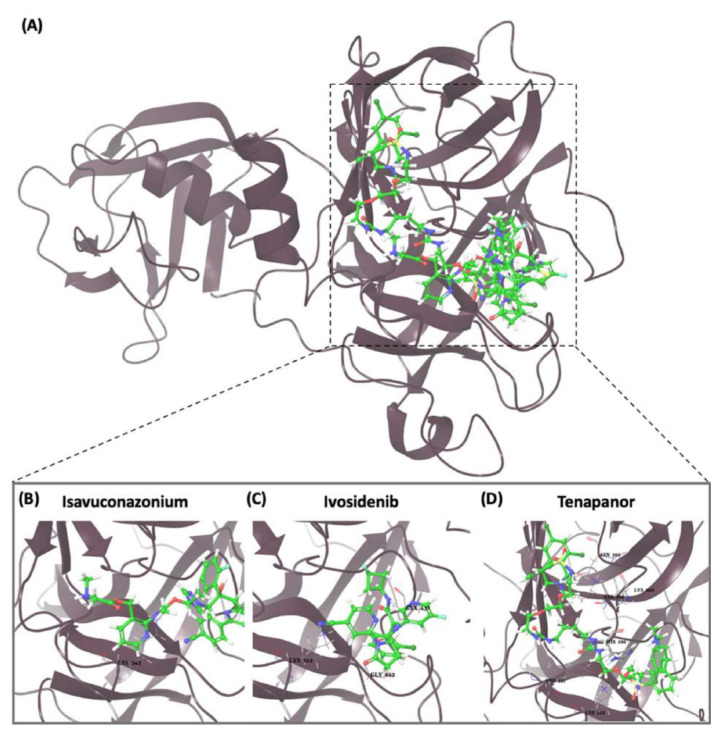
(A) The orientation of top-scored small molecules in the binding pocket of the TMPRSS2 protein during a nucleophilic addition reaction, involving (B) isavuconazonium, (C) ivosidenib, and (D) tenapanor.

The covalent docking study with the nucleophilic addition reaction was performed to investigate the interaction between various compounds and the TMPRSS2 protein. The docking scores and the residues involved in the interactions were retrieved for the top-ranked molecules (Figure 2). The results are summarized in Table 2. Among the top three compounds investigated, isavuconazonium had the lowest docking score of −7.612 kcal/mol, and MMGBSA was −69.43 kcal/mol, indicating a favorable binding affinity toward the TMPRSS2 protein. However, Figure S6 shows the interaction mainly involved LYS342 in both hydrogen and water bridge interactions, suggesting a relatively localized binding mode. Tenapanor, on the other hand, showed a slightly lower docking score of −6.063 kcal/mol with a MMGBSA of −85.8 kcal/mol but interacted with a broader range of residues, including HIS296 that had hydrogen and hydrophobic interactions, as shown in Figure S7. VAL298, LYS300, ASN303, TYR337, and LYS342 were involved in hydrogen and water bridge interactions. The involvement of multiple residues suggests a more extensive binding interface and potentially more stable interactions with the target protein. Ivosidenib had the highest docking score of −5.841 kcal/mol, and an MMGBSA value of −67.83 kcal/mol. Its interactions, as shown in Figure S8, were mediated by hydrogen bonding with CYS437 and GLY462 residues. LYS342 showed both hydrogen and water bridge interactions. It is noteworthy that LYS342 was a common interacting residue among all three compounds, suggesting its potential importance in the binding interactions of potential inhibitors with the TMPRSS2 protein.

Figure S9 shows the RMSD of backbone atoms for protein-ligand complexes over time for the three molecules: tenapanor, isavuconazonium, and ivosidenib, during a MD simulation with the nucleophilic addition reaction. For tenapanor (green), the RMSD values fluctuate around 4 to 6 Å for the majority of the simulation time, indicating relatively moderate conformational changes in the backbone atoms compared to the initial structure. Isavuconazonium (purple) had lower RMSD values, mostly ranging between 2 and 4 Å, suggesting smaller deviations from the initial structure and a more stable backbone conformation throughout the simulation. On the other hand, ivosidenib (blue) displays the highest RMSD values among the three molecules, with values reaching approximately 10 Å towards the end of the simulation (approximately 90 ns). This suggests that ivosidenib-complex undergoes larger conformational changes during the simulation, deviating more significantly from its initial structure.

TMPRSS2 is a key host protein that enables membrane fusion and subsequent viral entry (Manandhar et al., 2022). The nucleophilic addition reaction found the best candidates to inhibit this protein. The following top molecules emerged as potential inhibitors of TMPRSS2: tenapanor, isavuconazonium, and ivosidenib. Prominently, tenapanor, which is a small-molecule inhibitor approved for the treatment of irritable bowel syndrome with constipation (IBS-C) (Siddiqui and Cash, 2020), showed promising results in computational studies targeting TMPRSS2. Through virtual screening and molecular docking simulations, tenapanor was identified as a potential TMPRSS2 inhibitor, suggesting its potential repurposing as an antiviral agent against SARS-CoV-2. In addition, isavuconazonium, an antifungal medication primarily used in the treatment of invasive aspergillosis and mucormycosis (De et al., 2022), was investigated for its potential to inhibit TMPRSS2. Ullah et al. (2022) highlighted the role of isavuconazonium in COVID-19-associated invasive mold infections. Our study found favorable binding interactions with TMPRSS2, indicating its potential as a repurposed drug candidate for COVID-19 treatment. Ivosidenib is a small-molecule inhibitor approved for the treatment of relapsed or refractory acute myeloid leukemia (AML) (Gavillet et al., 2020). In this study, we identified ivosidenib as a potential TMPRSS2 inhibitor through covalent docking and MD simulations, suggesting its potential repurposing as an antiviral agent against SARS-CoV-2. While these computational findings are encouraging, further experimental validation and clinical trials are necessary to confirm the efficacy and safety of these compounds as TMPRSS2 inhibitors for COVID-19 treatment. Nevertheless, the repurposing of existing drugs could accelerate the development of effective COVID-19 therapeutics, highlighting the importance of such computational studies in the ongoing fight against the pandemic.

### 3.3. Potential Mpro protein inhibitors with the betalactam reaction

**Figure 3:**
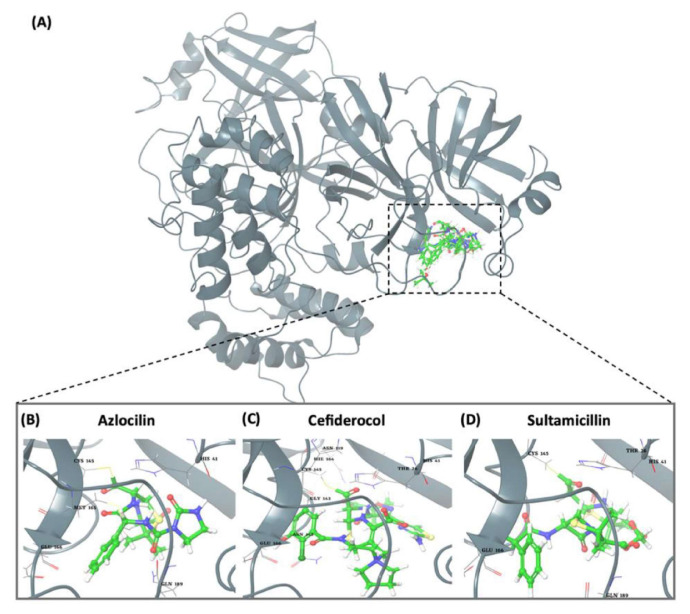
(A) The orientation of top-scored small molecules in the binding pocket of the Mpro protein during a betalactam reaction, involving (B) azlocillin, (C) cefiderocol, and (D) sultamicillin.

The covalent docking analysis with the betalactam reaction was performed to identify potential inhibitors targeting the Mpro protein (Figure 3). The docking scores and the residues involved in the interaction between each molecule and the Mpro protein are shown in Table 3. Azlocillin had the lowest docking score of −6.836 kcal/mol among the evaluated compounds, and a MMGBSA value of −66.07 kcal/mol. In Figure S10, the key residue involved in the hydrogen interaction between azlocillin and Mpro is HIS41. GLU166 and GLN189 had hydrogen bonds and water bridges. A hydrophobic interaction was observed with MET165, while CYS145 had hydrogen and hydrophobic interactions. The second-ranked molecule was cefiderocol, with a docking score of −6.385 kcal/mol and an MMGBSA score of −52.49 kcal/mol. Figure S11 shows the residues involved in both hydrogen and water bridge interactions with cefidecorol and Mpro: THR26, ASN119, ASN142, GLY143, HIS164, and GLU166. HIS41 and SER145 showed hydrogen and hydrophobic interactions. This diverse set of residues suggests that cefiderocol has favorable binding affinity. Finally, sultamicillin had a docking score of −5.883 kcal/mol and MMGBSA was −71.78 kcal/mol. In Figure S12, the residues implicated in the hydrophobic interaction with Mpro were CYS145 and water bridge interactions with THR26 and GLU166. HIS41 and GLN189 had hydrogen and water bridge interactions. Sultamicillin had a slightly higher docking score compared to azlocillin and cefiderocol, indicating a lower binding affinity. However, all three molecules did share some common interacting residues, such as HIS41, CYS145, and GLU166, suggesting a similar binding mode.

The RMSD of backbone atoms of protein-ligand complexes for the three molecules (azlocillin, cefiderocol, and sultamicillin) over a simulation time of 100 ns in the betalactam reaction system is shown in Figure S13. Azlocillin-complex (dark blue) had relatively stable RMSD values around 1 to 2.5 Å for most of the simulation time. However, there are occasional spikes in RMSD, indicating temporary deviations from the starting structure between 50 and 70 ns. Overall, the complex generally returns to a stable conformation. Cefiderocol-complex (light blue) shows less fluctuations in RMSD at approximately 2 Å. The RMSD values ranged from approximately 2 Å to 2.5 Å, suggesting a relatively stable degree of structural and conformational changes during the simulation. Sultamicillin-complex (green) displays a similar RMSD profile to cefiderocol, with fluctuations between 2 Å and 2.5 Å.

Among various reactions, two groups showed potential hit molecules targeting Mpro of SARS-CoV-2. The first group includes betalactam reactions involving azlocillin, cefiderocol, and sultamicillin that showed promising inhibitory activity against Mpro through computational studies. Azlocillin, a novel monobactam antibiotic, has shown promising results in computational studies targeting SARS-CoV-2 Mpro. This finding was corroborated by Pothineni et al. (2020) that aligns with our findings that identified azlocillin as a potential Mpro inhibitor through virtual screening and molecular docking simulations, suggesting its potential repurposing as an antiviral agent against COVID-19. Furthermore, cefiderocol, a novel siderophore cephalosporin antibiotic approved for the treatment of complicated urinary tract infections (cUTIs) and hospital-acquired or ventilator-associated bacterial pneumonia (HABP/VABP) (McCarthy, 2020), was also investigated for its potential to inhibit Mpro. A case study highlighted cefiderocol-containing regimens and cefiderocol + fosfomycin in combination for favorable survival rates (Russo et al., 2023), indicating its potential as a repurposed drug candidate for COVID-19 treatment. Additionally, sultamicillin, a combination of ampicillin and sulbactam, has shown promising results in the treatment of upper respiratory tract infections (Ferreira et al., 2006). In this study, we identified sultamicillin as a potential Mpro inhibitor through virtual screening and molecular docking simulations, suggesting its potential repurposing as an antiviral agent against COVID-19.

### 3.4. Exploring betalactam reaction candidates for ACE2 protein inhibition via covalent docking analysis

**Figure 4:**
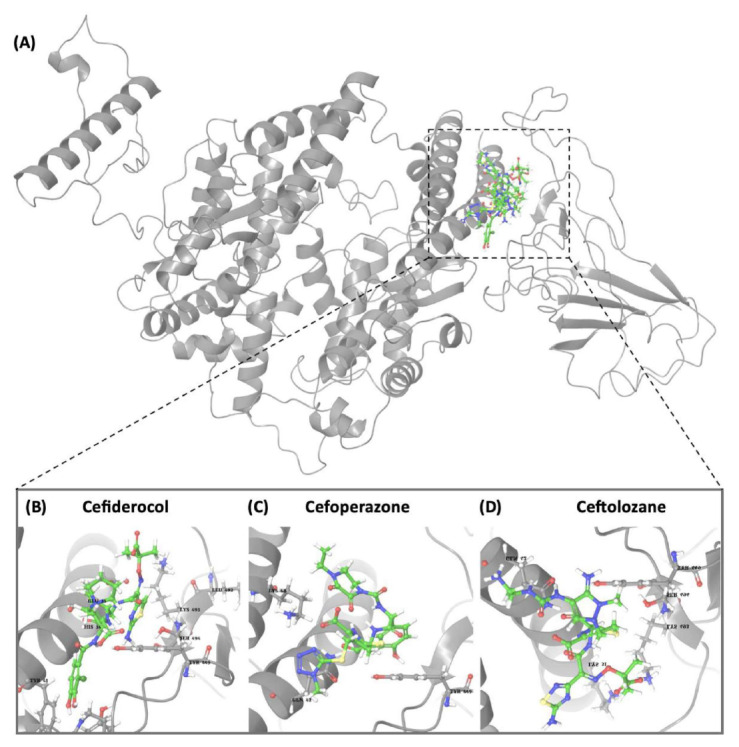
(A) The orientation of top-scored small molecules in the binding pocket of the ACE2 protein during betalactam reaction, involving (B) cefiderocol, (C) cefoperazone, and (D) ceftolozane.

The covalent docking study involving the betalactam reaction with the S/ACE2 protein complex revealed several promising compounds with favorable docking scores and key interaction residues summarized in Table 4 and Figure 4. The analysis identified the top three compounds as cefiderocol, ceftolozane, and cefoperazone, each showing potential binding affinities to the S/ACE2 protein complex. Cefiderocol had the lowest docking score of −4.781 kcal/mol with a MMGBSA value of −180.25 kcal/mol, indicating a strong binding affinity to the ACE2 protein. The key residues illustrated in Figure S14 that were involved in the hydrogen bonding interactions between cefiderocol and the S/ACE2 protein complex were LYS493, ARG498, and TYR501 on the B chain (S protein), along with a water bridge interaction with SER494 on the B chain. Additionally, a hydrophobic interaction was observed with the TYR41 residue on the A chain (ACE2). Furthermore, TYR449 on the B chain displayed diverse interactions, including hydrogen bonding, hydrophobic interactions, and water bridge interactions. Water bridges were also observed with HIS34, GLU35, and GLN42 on the A chain and LEU492 on the B chain. Ceftolozane had a docking score of −4.227 kcal/mol with a MMGBSA score of −121.79 kcal/mol, indicating favorable binding to the active site of the S/ACE2 protein complex. Figure S15 illustrates the key residues involved in facilitating the binding of ceftolozane, including hydrogen bonding and water bridge interactions with GLN42 on the A chain, and PHE490, LYS493, and SER494 on the B chain. Additionally, LYS31 and LYS68 on the A chain, and TYR449 and ARG498 on the B chain had ionic, hydrophobic, hydrogen bonding, and water bridge interactions. Cefoperazone had a docking score of −3.840 kcal/mol, and the determined MMGBSA was −139.51 kcal/mol, suggesting a potential binding affinity to the S/ACE2 protein complex. The residues involved in the interactions had diverse interaction types, as shown in Figure S16, including hydrogen bonding, water bridges, and hydrophobic interactions. Notably, TYR449 on the B chain showed hydrophobic interactions in addition to other interaction types, while LYS68 on the A chain had ionic interactions. This covalent docking study provides valuable insights into the potential repurposing of small molecules via betalactam reactions as therapeutic agents for COVID-19 by targeting the S/ACE2 protein complex, which serves as the primary entry point for the SARS-CoV-2 virus into human cells.

The RMSD of backbone atoms of protein-ligand complexes for the three molecules cefiderocol, cefoperazone, and ceftolozane during the 100 ns MD simulations is shown in Figure S17. Cefiderocol-complex had relatively small RMSD values of around 2 to 3 Å for most of the simulation period, indicating less deviation from the starting point. In contrast, cefoperazone-complex displayed larger RMSD fluctuations, ranging from approximately 2 to 6 Å, suggesting more significant structural deviations over time. Ceftolozane-complex had an intermediate RMSD profile, with values typically 3–5 Å, indicating moderate structural changes compared to the other two molecules. These differences in RMSD magnitudes suggest that the three complexes experienced varying degrees of structural flexibility or conformational changes during the simulated reaction process, potentially influenced by their respective chemical structures and properties. Based on the RMSD data, cefiderocol appeared to be the most structurally stable molecule of the three (Figure 4).

In our study, we identified promising candidate molecules targeting ACE2, a functional receptor for the SARS-CoV-2 S protein, facilitating viral entry into host cells. Inhibiting ACE2 could disrupt this process and prevent viral entry, making it an attractive target for potential antiviral therapies (Hoffmann et al., 2020). The candidate molecules were cefiderocol, cefoperazone, and ceftolozane that emerged as potential ACE2 inhibitors through computational studies. Cefiderocol, a novel siderophore cephalosporin antibiotic approved for the treatment of complicated urinary tract infections (cUTIs) and hospital-acquired or ventilator-associated bacterial pneumonia (HABP/VABP) (McCarthy, 2020), showed promising results in computational studies targeting ACE2. Furthermore, cefoperazone is a third-generation cephalosporin antibiotic primarily used in the treatment of urinary and respiratory infections (Xin et al., 2013). In our study, we showed the potential of cefoperazone to inhibit ACE2, indicating its potential as a repurposed drug candidate for COVID-19 treatment. Additionally, ceftolozane, a novel cephalosporin antibiotic approved for the treatment of complicated intraabdominal and urinary tract infections (Zhanel et al., 2014), also showed promising results in computational studies targeting ACE2. The potential ACE2 inhibitor was identified through virtual screening and molecular docking simulations, suggesting its potential repurposing as an antiviral agent against COVID-19.

Targeting ACE2, the primary cellular receptor for SARS-CoV-2, represents a promising strategy to block viral infection by preventing the pathogen’s entry into host cells. This approach offers significant advantages as ACE2 serves as the critical gateway for viral attachment and subsequent cellular invasion. However, this therapeutic strategy faces important biological challenges. ACE2 fulfills essential physiological roles throughout multiple organ systems, including blood pressure regulation, pulmonary function maintenance, and renal homeostasis. Consequently, the optimal intervention would achieve selective modulation rather than complete inhibition of the viral-binding capacity of ACE2 while maintaining its beneficial physiological functions intact (Jia et al., 2021). To develop such precisely targeted interventions, additional research is essential to evaluate the ability of our candidate inhibitors to effectively modulate ACE2 activity in a balanced manner.

## 4. Conclusion

In conclusion, this study has identified several promising FDA-approved drugs that could be repurposed as COVID-19 therapeutics by targeting crucial viral proteins. These drugs include bremelanotide, lanreotide, histrelin, and leuprolide for RdRp inhibition; azlocillin, cefiderocol, and sultamicillin for Mpro inhibition; tenapanor, isavuconazonium, and ivosidenib targeting TMPRSS2; and cefiderocol, cefoperazone, and ceftolozane as potential ACE2 inhibitors. These findings provide valuable insights into the repurposing of existing drugs as potential COVID-19 therapeutics. The repurposing of FDA-approved drugs offers the potential to expedite the availability of effective treatments for COVID-19 patients. This approach can provide faster and more affordable treatment options compared to the time-consuming and costly process of developing entirely new drugs. Moreover, the drugs identified in this study have already undergone extensive clinical trials and have established safety profiles that significantly reduce the risks typically associated with drug development. As a result, these drugs could be transitioned into clinical trials for COVID-19 treatment faster than novel drug candidates, potentially offering timely solutions to the pandemic.

However, while computational predictions suggest promising candidates, it is important to emphasize that experimental validation of these findings is crucial to confirm their efficacy against COVID-19. Due to resource limitations, this study did not include experimental validation. Nevertheless, we propose that future research should focus on in vitro and in vivo experiments to assess the therapeutic potential of these repurposed drugs. Such validation can be performed to explore the effects of the drugs on animal models to evaluate therapeutic efficacy and safety in vivo. Together with in vitro and in vivo experiments, the necessity to perform off target covalent inhibition should be considered. A lack of in vitro and in vivo experimental validation, and off target covalent inhibition assessment, are limitations of the current study. If these drugs show promise in preclinical studies, they could then be prioritized for clinical trials specific to COVID-19. While further research is required to establish the clinical efficacy of these drugs against COVID-19, the findings in this study provide a solid foundation for future investigation and offer a potential pathway to accelerate the development of effective treatments against this disease.

## Supporting information

Supplementary File was deposited to the APERTA database, accessible at https://aperta.ulakbim.gov.tr/record/285984



## Figures and Tables

**Table 1:** Docking and MMGBSA scores, and interaction residues for the top four molecules in the nucleophilic addition to a double bond reaction with RdRp.

Nucleophilic addition to a double bond reaction				
Compound	2D structure	Docking (kcal/mol)	MMGBSA (kcal/mol)	Interaction residues
Bremelanotide	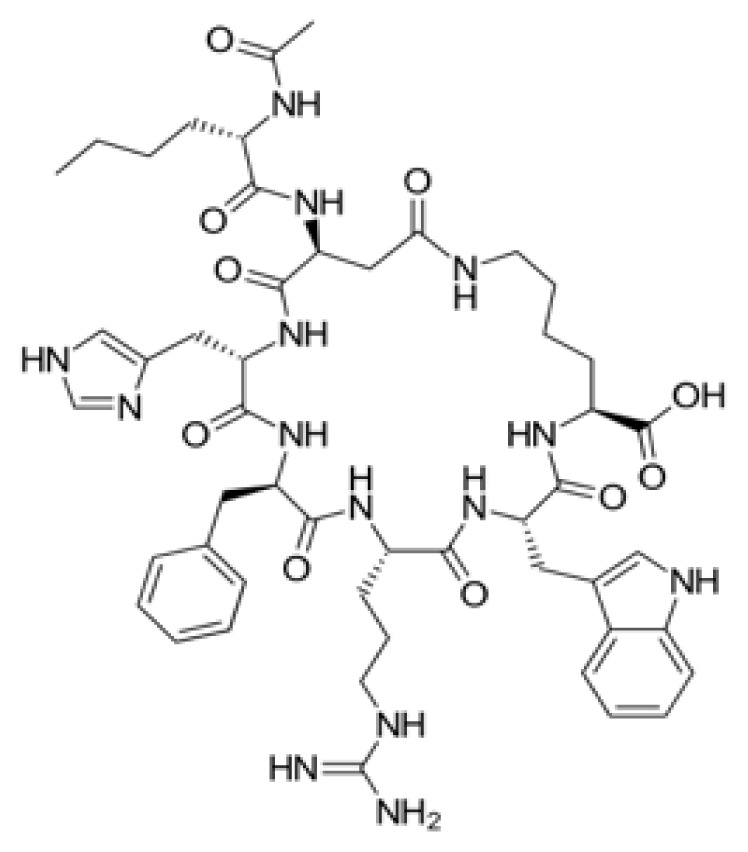	−8.617	−96.6	ASP452, LYS545, ARG555, THR556, ASP618, TYR619, CYS622, ASP623, SER759, ASP760, ASP761, GLU811, SER814
Lanreotide	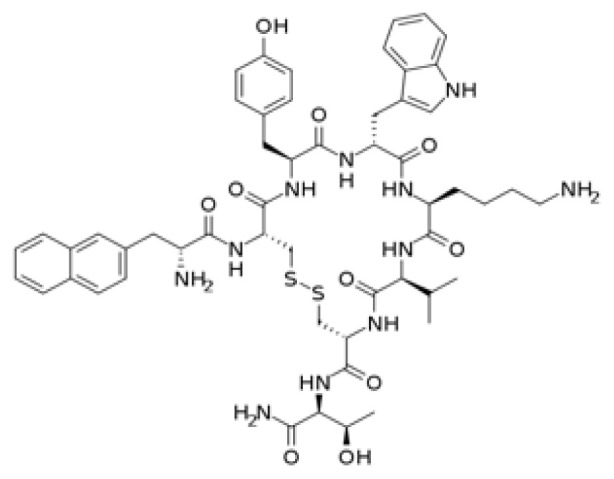	−7.714	−87.4	ARG555, THR556, ALA558, CYS622, ARG624, SER682, ASP760, SER814
Histrelin	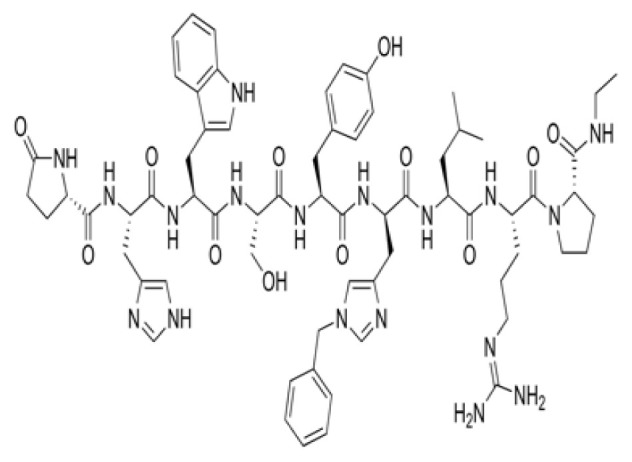	−6.465	−94.5	ASP452, ALA554, THR556, TYR619, CYS622, ASP623, SER759, ASP760
Leuprolide	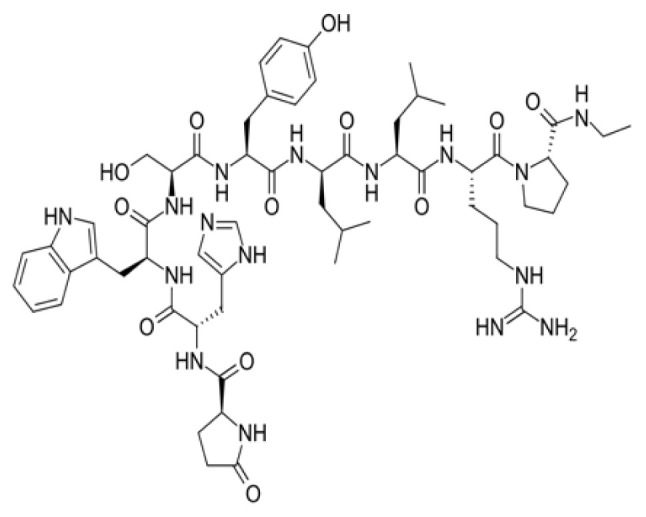	−5.788	−140.2	ARG555, ALA558, ARG569, GLY590, CYS622, ASP623, THR680, SER682, ALA685, ALA688, TYR689, SER759, ASP760, ASP761

**Table 2:** Docking and MMGBSA scores, and interaction residues for the top three molecules in the nucleophilic addition reaction with the TMPRSS2 protein.

Nucleophilic addition reaction				
Compound	2D structure	Docking(kcal/mol)	MMGBSA (kcal/mol)	Interaction residues
Isavuconazonium	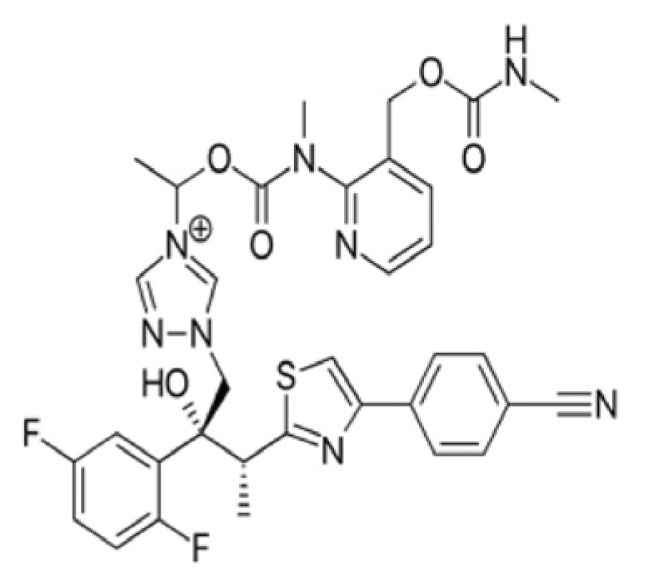	−7.612	−69.43	LYS342
Tenapanor	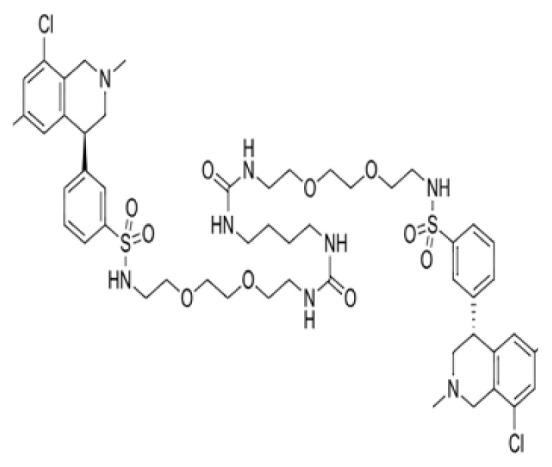	−6.063	−85.8	HIS296, VAL298, LYS300, ASN303, TYR337, LYS342
Ivosidenib	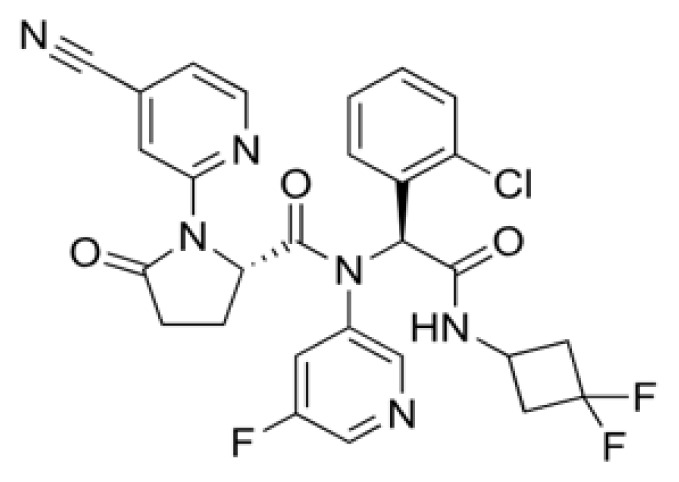	−5.841	−67.83	LYS342, CYS437, GLY462

**Table 3:** Docking and MMGBSA scores, and interaction residues for the top three molecules in the betalactam reaction with the Mpro protein.

Betalactam reaction				
Compound	2D structure	Docking (kcal/mol)	MMGBSA (kcal/mol)	Interaction residues
Azlocillin	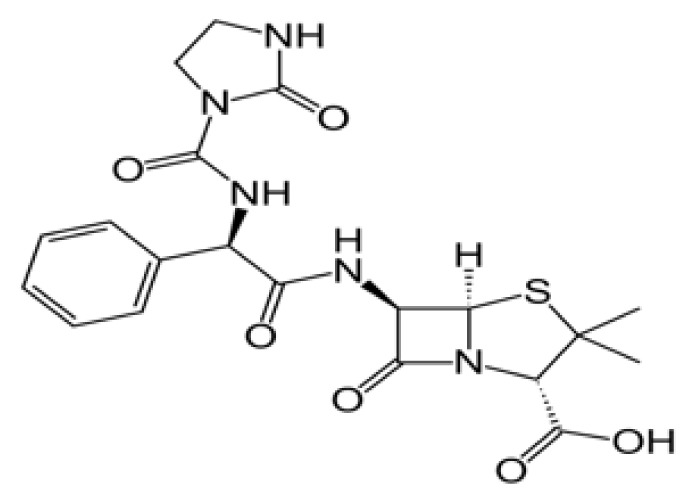	−6.836	−66.07	HIS41, CYS145, MET165, GLU166, GLN189
Cefiderecol	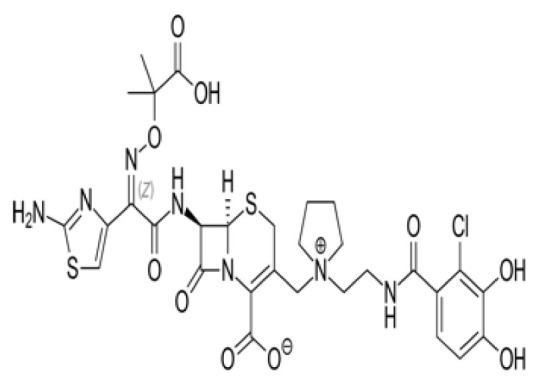	−6.385	−52.49	THR26, HIS41, ASN119, ASN142, GLY143, SER145, HIS164, GLU166
Sultamicillin	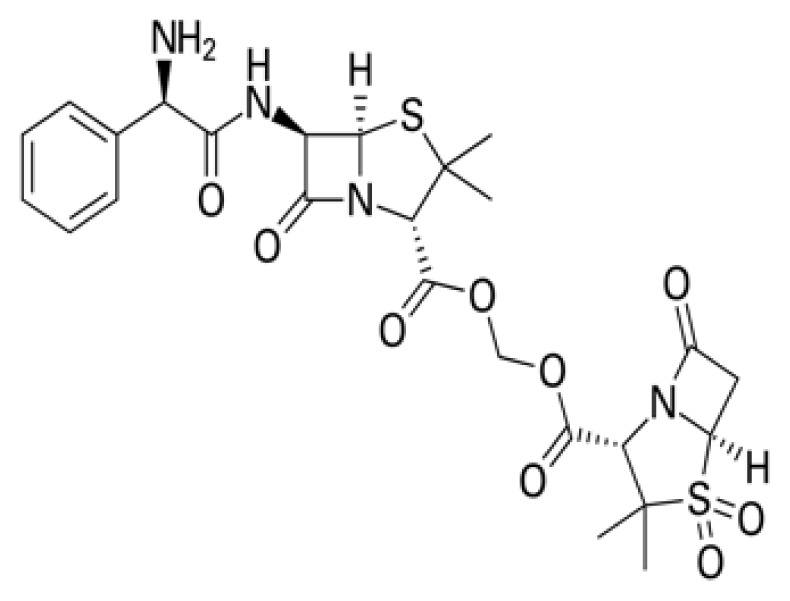	−5.883	−71.78	THR26, HIS41, CYS145, GLU166, GLN189

**Table 4:** Docking and MMGBSA scores, and interaction residues for the top three molecules in the betalactam reaction with the S/ACE2 protein complex.

Betalactam reaction				
Compound	2D structure	Docking (kcal/mol)	MMGBSA (kcal/mol)	Interaction residues
Cefiderocol	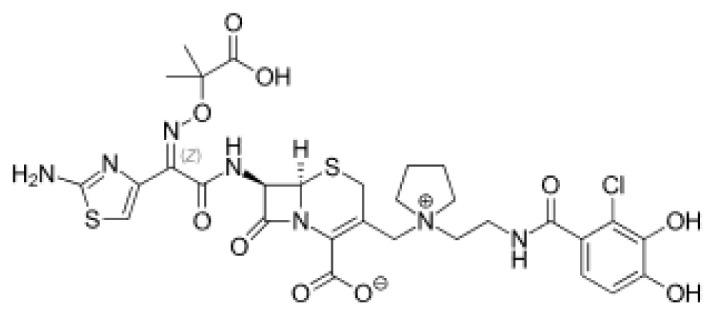	−4.781	−180.25	A:HIS34, A:GLU35 A:TYR41, A:GLN42, A:TYR449, B:LEU492, B:LYS493, B:SER494, B:ARG498, B:TYR501,
Ceftolozane	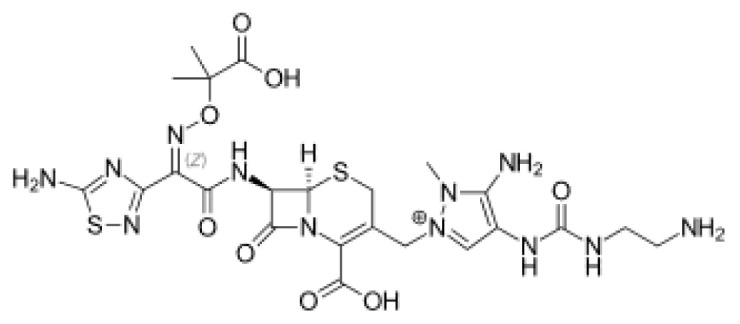	−4.227	−121.794	A:LYS31, A:GLN42, B:TYR449, B:PHE490, B:LYS493, B:SER494, B:ARG498
Cefoperazone	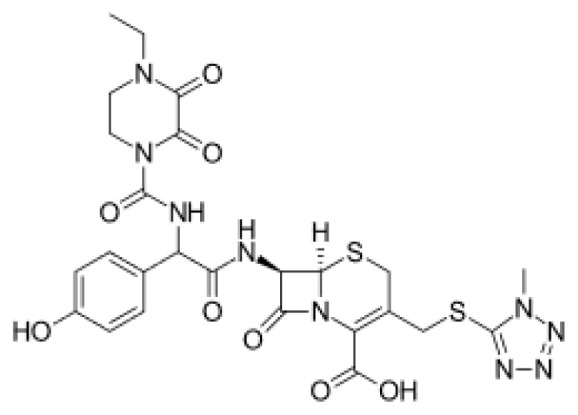	−3.840	−139.519	A:LYS31, A:GLN42, B:TYR449, B:PHE490, B:LYS493, B:SER494, B:ARG498

## Footnotes

1 Schrödinger Suite. Schrödinger, LLC; New York, NY: **2012**.
