# Supplementary material for: FDA-approved drugs as potential covalent inhibitors of key SARS-CoV-2 proteins: an in silico approach
Source: Turk J Biol. 2025 Apr 7;49(3):233–46. doi: 10.55730/1300-0152.2741 (PMC12266346; doi:10.55730/1300-0152.2741)
Supplement: Supplementary file 1 [file tjb-49-03-233_SupplementaryData.docx]

**SUPPORTING INFORMATIONS**

**Supplementary Figures**


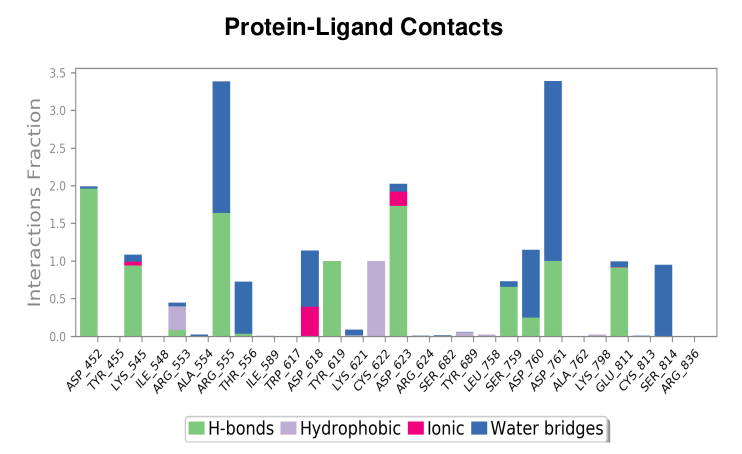


**Figure S1.** The bar chart illustrates the nucleophilic addition to double bond reaction formed between the drug bremelanotide and the RdRP protein, highlighting hydrogen bonds, hydrophobic interactions, ionic bonds, and water bridges during 100 ns molecular simulations.
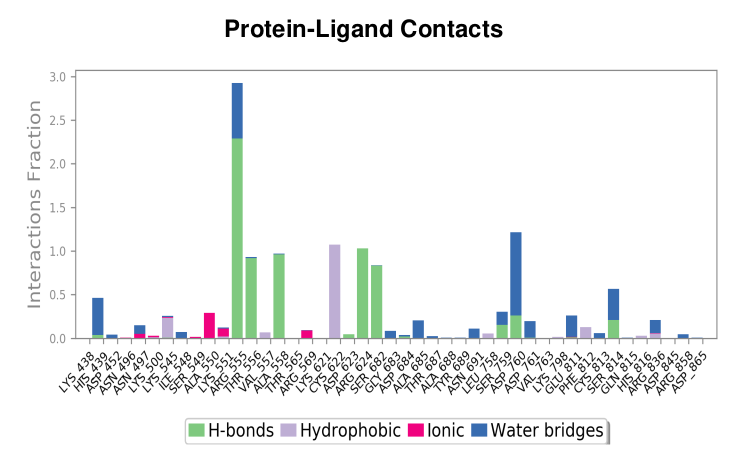


**Figure S2.** The bar chart illustrates the nucleophilic addition to double bond reaction formed between the drug lanreotide and the RdRP protein, highlighting hydrogen bonds, hydrophobic interactions, ionic bonds, and water bridges during 100 ns molecular simulations.


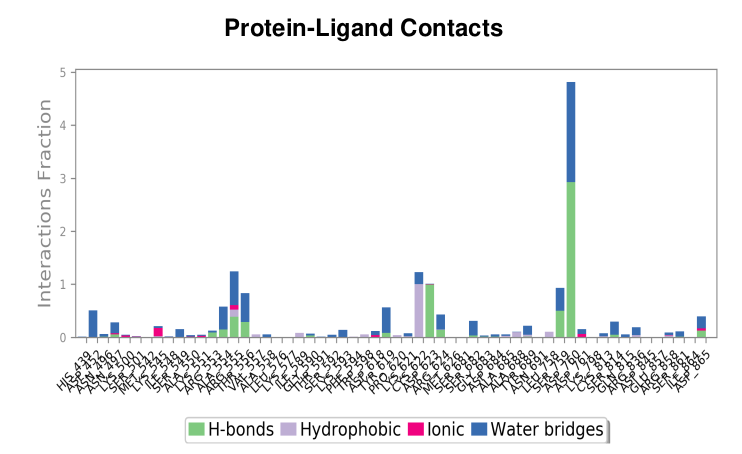


**Figure S3.** The bar chart illustrates the nucleophilic addition to double bond reaction formed between the drug histrelin and the RdRP protein, highlighting hydrogen bonds, hydrophobic interactions, ionic bonds, and water bridges during 100 ns molecular simulations.


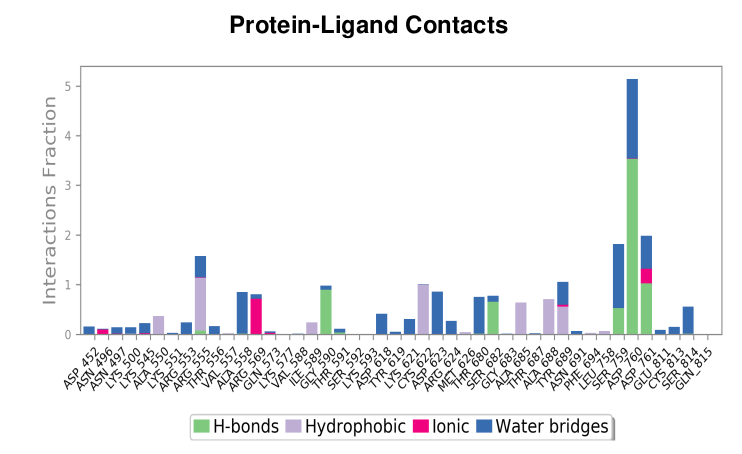


**Figure S4.** The bar chart illustrates the nucleophilic addition to double bond reaction formed between the drug leuprolide and the RdRP protein, highlighting hydrogen bonds, hydrophobic interactions, ionic bonds, and water bridges during 100 ns molecular simulations.

**Figure S5.** RMSD of the carbon backbone atoms of the RNA-dependent RNA polymerase (RdRp) protein with the top three compound complexes with nucleophilic addition to a bond reaction over 100 ns molecular dynamics (MD) simulations.


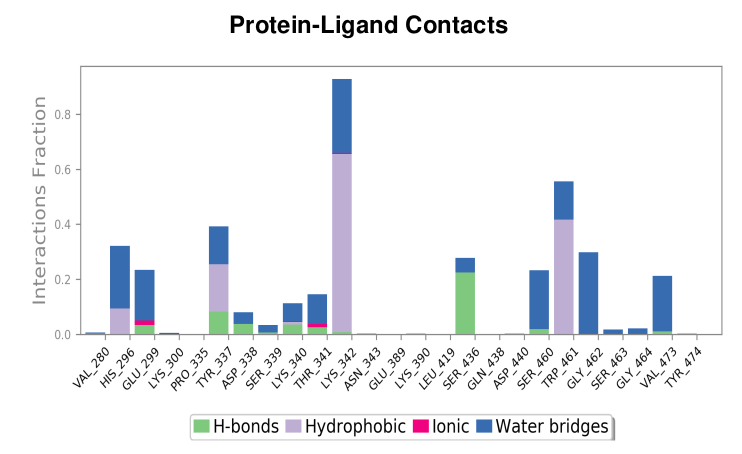


**Figure S6.** The bar chart illustrates the nuclephilic addition reaction formed between the drug isavuconazonium and the TMPRSS2 protein, highlighting hydrogen bonds, hydrophobic interactions, ionic bonds, and water bridges during 100 ns molecular simulations.


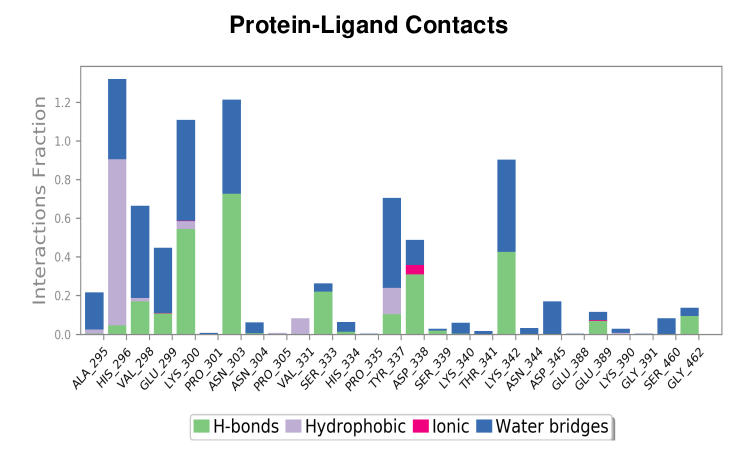


**Figure S7.** The bar chart illustrates the nuclephilic addition reaction formed between the drug tenapanor and the TMPRSS2 protein, highlighting hydrogen bonds, hydrophobic interactions, ionic bonds, and water bridges during 100 ns molecular simulations.


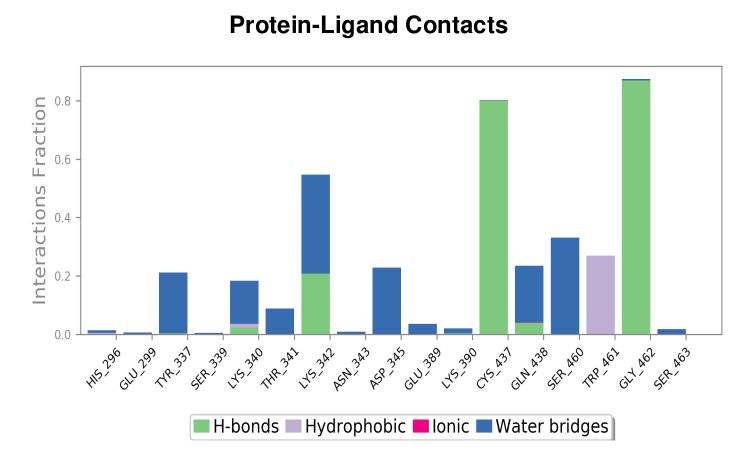


**Figure S8.** The bar chart illustrates the nuclephilic addition reaction formed between the drug ivosidenib and the TMPRSS2 protein, highlighting hydrogen bonds, hydrophobic interactions, ionic bonds, and water bridges during 100 ns molecular simulations.

**Figure S9.** RMSD of the carbon backbone atoms of the transmembrane serine protease 2 (TMPRSS2) protein with the top three compound complexes with nucleophilic addition reaction over 100 ns molecular dynamics (MD) simulations.


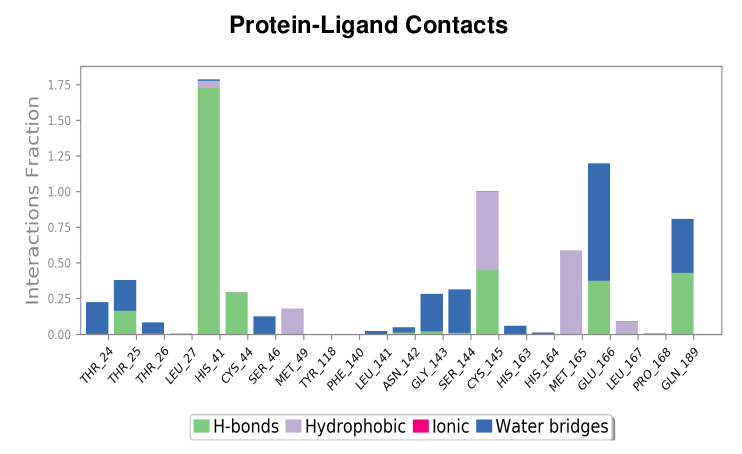


**Figure S10.** The bar chart illustrates the betalactam reaction formed between the drug azlocillin and the Mpro protein, highlighting hydrogen bonds, hydrophobic interactions, ionic bonds, and water bridges during 100 ns molecular simulations.


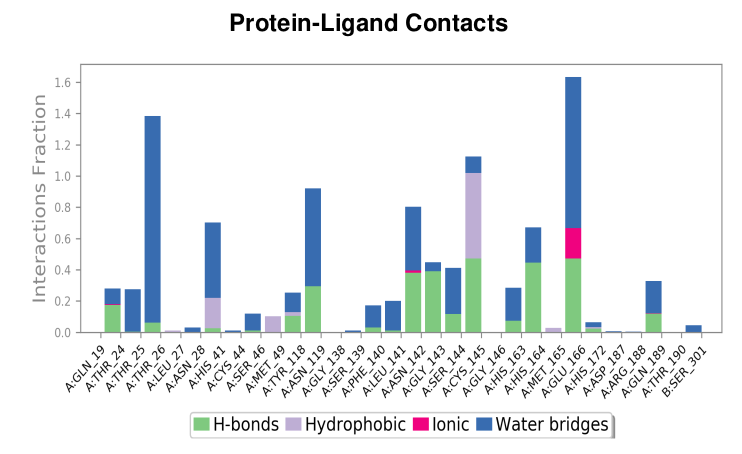


**Figure S11.** The bar chart illustrates the betalactam reaction formed between the drug cefiderecol and the Mpro protein, highlighting hydrogen bonds, hydrophobic interactions, ionic bonds, and ater bridges during 100 ns molecular simulations.


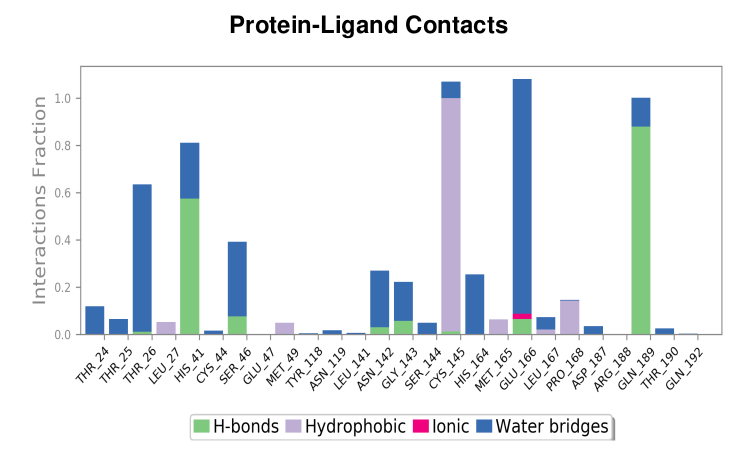


**Figure S12.** The bar chart illustrates the betalactam reaction formed between the drug sultamicillin and the Mpro protein, highlighting hydrogen bonds, hydrophobic interactions, ionic bonds, and water bridges during 100 ns molecular simulations.

**Figure S13.** RMSD of the carbon backbone atoms of the **main protease (Mpro**2) protein with the top three compound complexes with betalactam reaction over 100 ns molecular dynamics (MD) simulations.


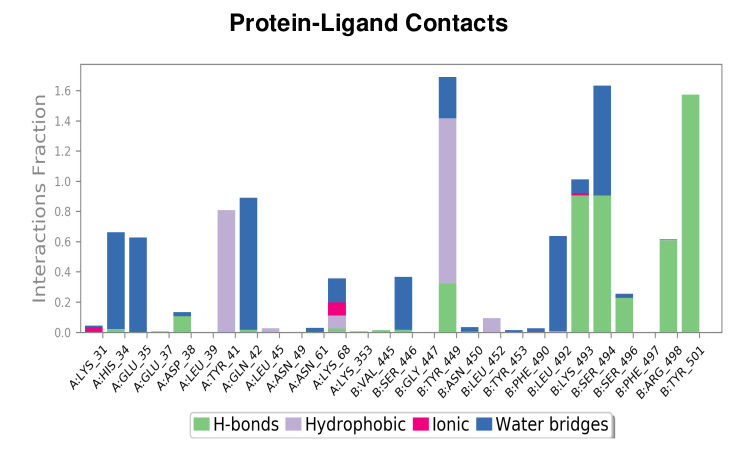


**Figure S14.** The bar chart illustrates the betalactam reaction formed between the drug cefiderocol and the Ace2 protein, highlighting hydrogen bonds, hydrophobic interactions, ionic bonds, and water bridges during 100 ns molecular simulations.


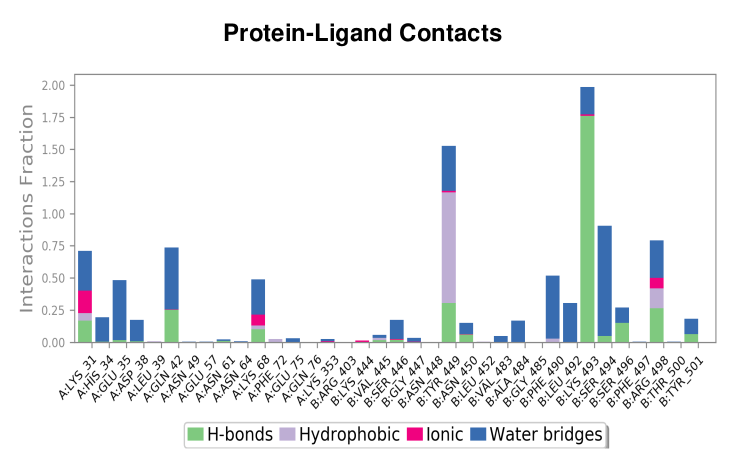


**Figure S15.** The bar chart illustrates the betalactam reaction formed between the drug ceftolozane and the Ace2 protein, highlighting hydrogen bonds, hydrophobic interactions, ionic bonds, and water bridges during 100 ns molecular simulations.


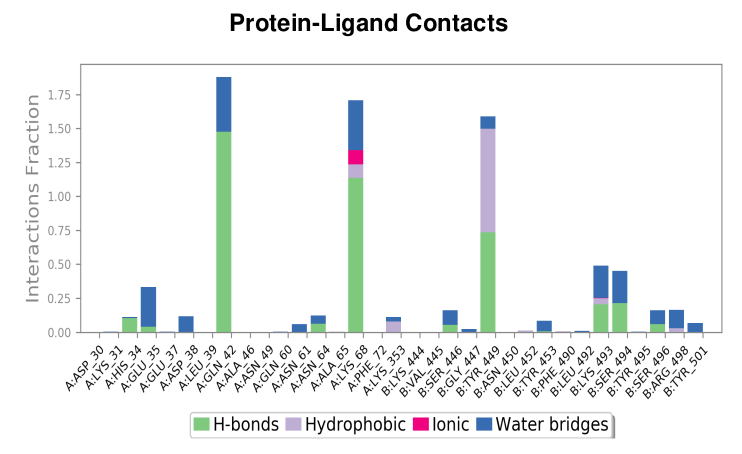


**Figure S16.** The bar chart illustrates the betalactam reaction formed between the drug cefoperazone and the Ace2 protein, highlighting hydrogen bonds, hydrophobic interactions, ionic bonds, and water bridges during 100 ns molecular simulations.

**Figure S17.** RMSD of the carbon backbone atoms of angiotensin-converting enzyme 2 **(Ace**2) protein with the top three compound complexes with betalactam reaction over 100 ns molecular dynamics (MD) simulations.
